# Supplementary material for: Dietary fatty acids fine-tune Piezo1 mechanical response
Source: Nat Commun. 2019 Mar 13;10:1200. doi: 10.1038/s41467-019-09055-7 (PMC6416271; doi:10.1038/s41467-019-09055-7)
Supplement: Supplementary file 2 — Supplementary Information [file 41467_2019_9055_MOESM2_ESM.docx]

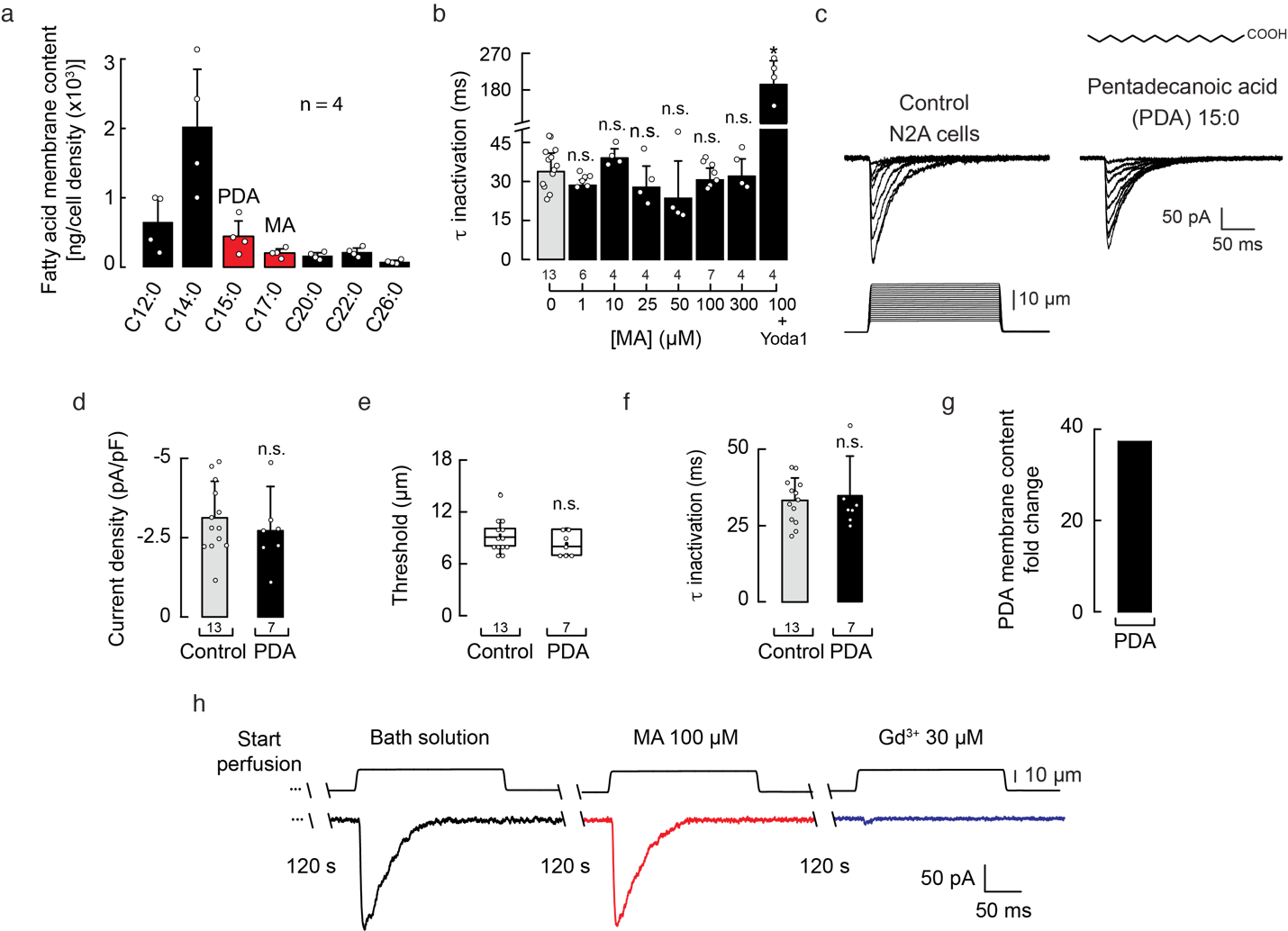


**Supplementary figure 1. Related to figure 1.**

**a** Saturated fatty acid content in N2A cells, as determined by LC-MS. C12:0, dodecanoic acid; C14:0, myristic acid; C15:0, pentadecanoic acid (PDA); C17:0, heptadecanoic acid (margaric acid, MA); C20:0, arachidic acid; C22:0, docosanoic acid; and C26:0, hexacosanoic acid. Bars are mean ± SD. n is denoted above the graph.

**b** Piezo1 time constants of inactivation elicited by maximum displacement (-60 mV) of control, MA-treated N2A cells, and MA (100 μM)-treated N2A cells + Yoda1 (15 μM). Bars are mean ± SD. n is denoted above the *x-*axis. Kruskal-Wallis and Dunn’s multiple comparisons test.

**c** Representative whole-cell patch-clamp recordings (at -60 mV) of control and PDA (100 μM)-treated N2A cells elicited by mechanical stimulation.

**d** Piezo1 current densities elicited by maximum displacement of control and PDA (100 μM)-treated N2A cells. Bars are mean ± SD. n is denoted inside bars. Unpaired t-test.

**e** Boxplots show the mean, median, and the 75^th^ to 25^th^ percentiles of the displacement thresholds required to elicit Piezo1 currents of control and PDA (100 μM)-treated N2A cells. n is denoted above the *x*-axis. Unpaired t-test.

**f** Piezo1 time constants of inactivation elicited by maximum displacement of control and PDA (100 μM)-treated N2A cells. Bars are mean ± SD. n is denoted above the *x*-axis. Unpaired t-test.

**g** PDA membrane content fold change in N2A cells treated with PDA 100 µM for 18 h, as determined by LC-MS.

**h** Representative traces of N2A cells Piezo1 currents after perfusing for 120 s bath solution (black), MA (red), and Gd^3+^ (blue) consecutively. Gd^3+^ was used as control for the perfusion. Traces were obtained from the same cell.

Asterisks indicate values significantly different from control (^∗^p < 0.05) and n.s. indicates not significantly different from the control.

**
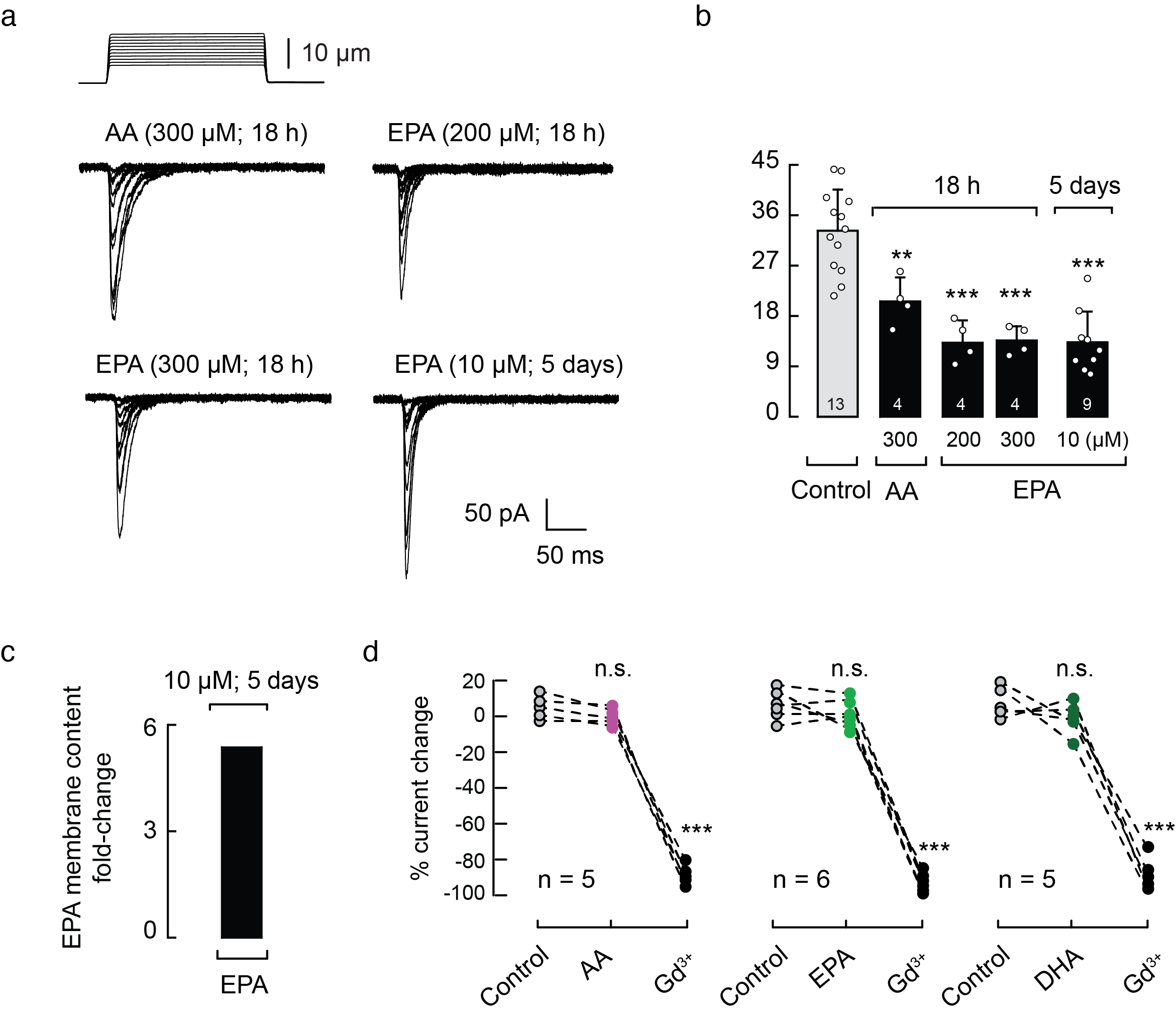
**

**Supplementary figure 2. Related to figure 2.**

**a** Representative whole-cell patch-clamp recordings (at -60 mV) of AA (300 μM) and EPA (200, 300 μM for 18h) and EPA (10 μM each day for five days)-treated N2A elicited by mechanical stimulation.

**b** Piezo1 time constants of inactivation elicited by maximum displacement of control, AA (300 μM), EPA (200, 300 μM; 18h) and EPA (10 μM each day for five days)-treated N2A cells. Bars are mean ± SD. n is denoted inside bars. One-way ANOVA and Bonferroni test.

**c** EPA membrane content fold change in EPA (10 µM each day for five days)-treated N2A cells, as determined by LC-MS.

**d** Current changes of N2A cells perfused for 120 s with bath solution, a PUFA (AA, EPA, and DHA; 100 μM), and Gd^3+^ (30 μM) consecutively. Gd^3+^ was used as control for the perfusion. Data samples are paired. Repeated measures analysis of variance and Bonferroni test. n is denoted above the *x*-axis

Asterisks indicate values significantly different from control (^∗∗∗^p < 0.001 and ^**^p < 0.01) and n.s. indicates not significantly different from the control.

**
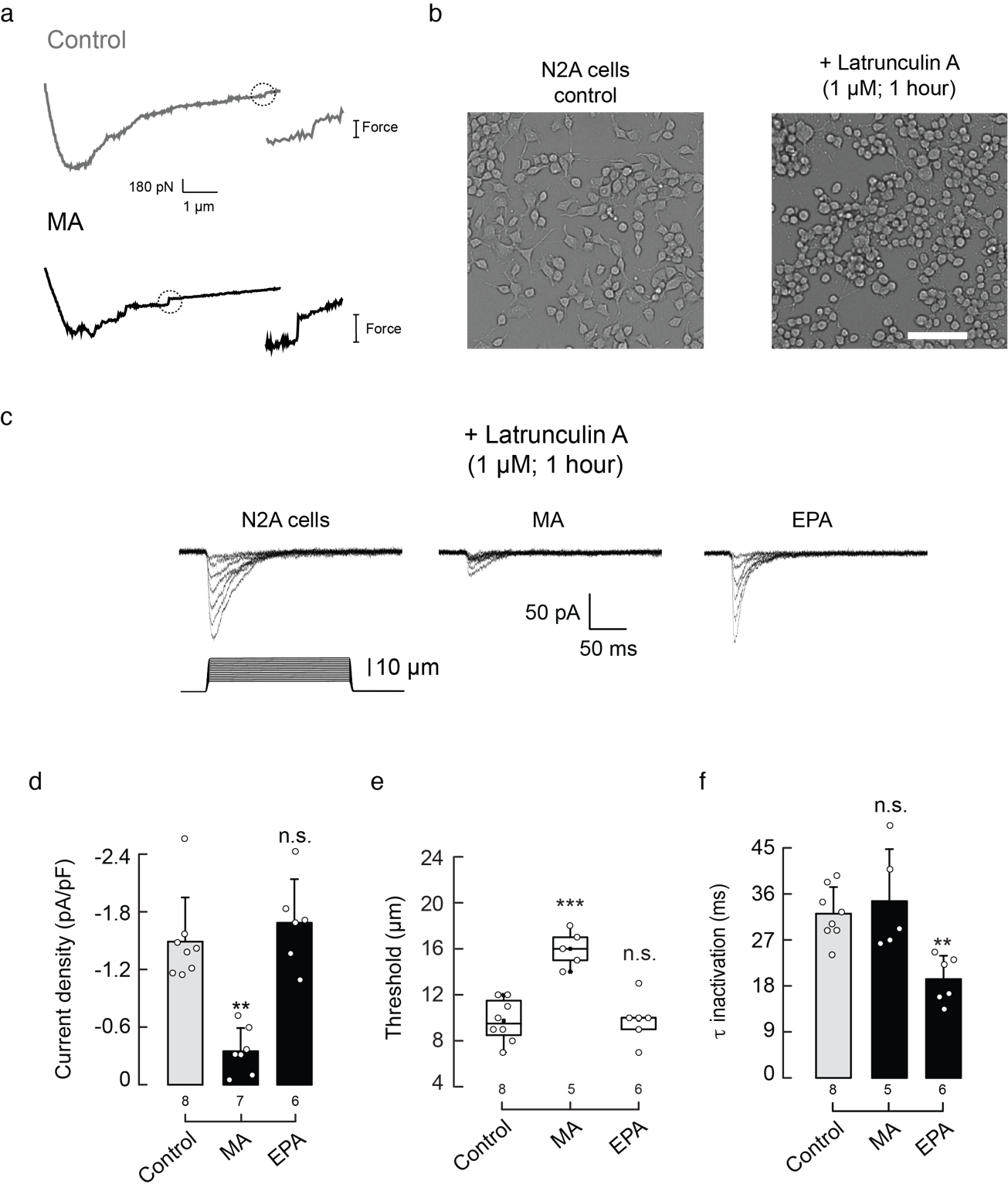
**

**Supplementary figure 3. Related to figure 3.**

**a** Representative force-distance traces acquired at 40 µm/s for control and MA (100 µM) N2A-treated cells.

**b** Representative micrographs of control and latrunculin A-treated (1 μM; 1 h)-N2A cells. White bar represents 100 μm.

**c** Representative whole-cell patch-clamp recordings (at -60 mV) elicited by mechanical stimulation of latrunculin A-treated (1 μM; 1 h)-N2A cells with and without MA (100 μM) and EPA (100 μM) supplementation.

**d** Current densities elicited by maximum displacement of latrunculin A (1 μM; 1 h)-treated N2A cells with and without MA (100 μM) and EPA (100 μM) supplementation. Bars are mean ± SD. Kruskal-Wallis and Dunn’s multiple comparisons test.

**e** Boxplots show the mean, median, and the 75^th^ to 25^th^ percentiles of the displacement threshold required to elicit Piezo1 currents of control and latrunculin A (1 μM; 1 h)-treated N2A cells with and without MA (100 μM) and EPA (100 μM) supplementation. One-way ANOVA and Bonferroni test.

**f** Piezo1 time constants of inactivation elicited by maximum displacement of latrunculin A (1 μM; 1 h)-treated N2A cells with and without MA (100 μM) and EPA (100 μM) supplementation. Bars are mean ± SD. One-way ANOVA and Bonferroni test.

Asterisks indicate values significantly different from control (^∗∗∗^p < 0.001 and ^∗∗^p < 0.01) and n.s. indicates not significantly different from the control. n is denoted above the *x*-axes

**
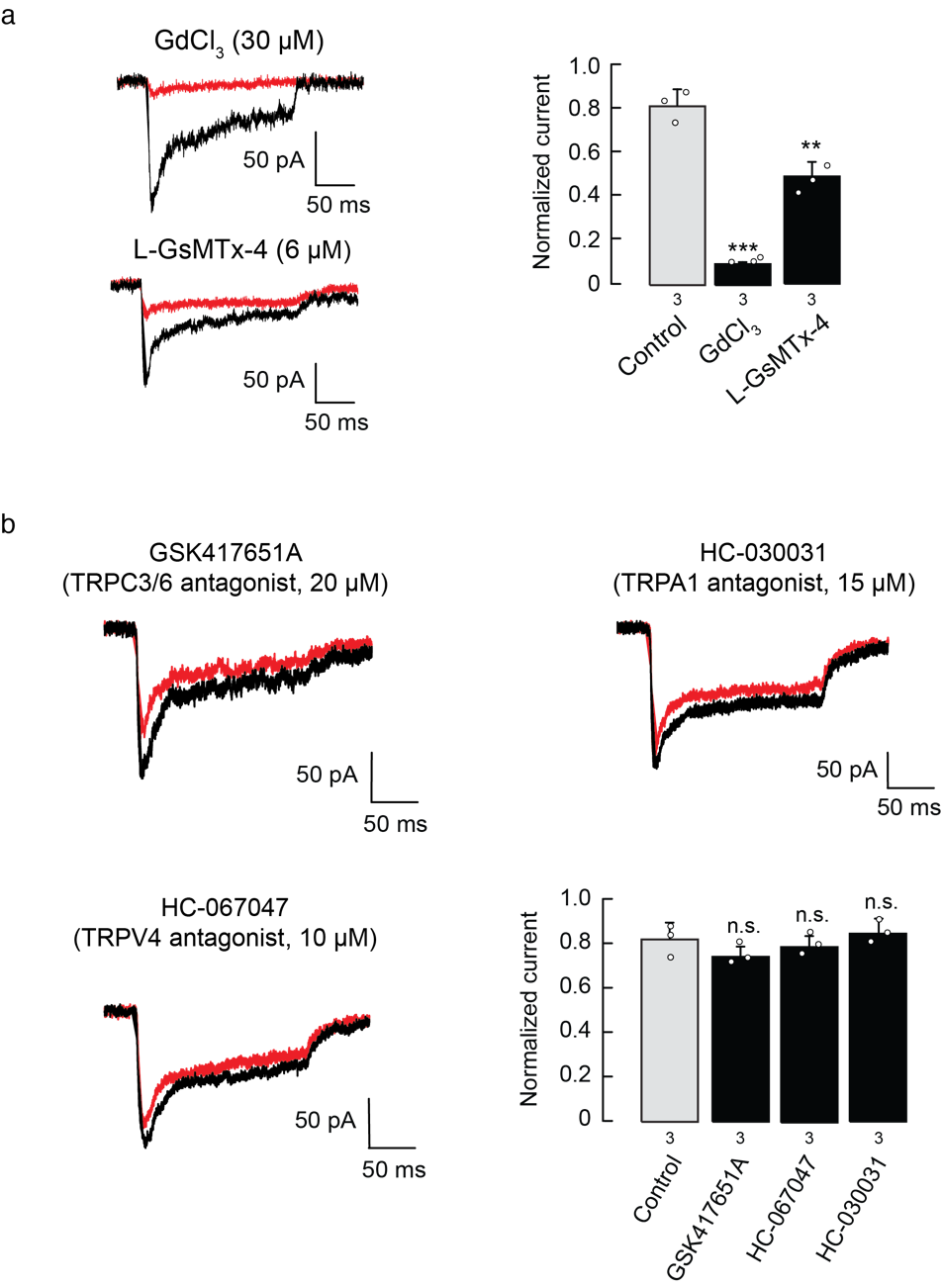
**

**Supplementary figure 4. Related to figure 4.**

**a** Left: Representative whole-cell patch-clamp recordings (at -60 mV) of HMVEC Piezo1 currents inhibited by GdCl_3_ (30µM) and L-GsMTx-4 (6 µM) while mechanically stimulating the cell. Right: Bar graph displaying Piezo1 inhibition by GdCl_3_ and L-GsMTx-4. Bars are mean ± SD. One-way ANOVA and Bonferroni test.

**b** Representative whole-cell patch-clamp recordings (at -60 mV) of HMVEC Piezo1 currents perfused with different TRP channel antagonists while mechanically stimulating the cell. Bottom: Bar graph displaying Piezo1 currents in the presence of TRP channel inhibitors. Bars are mean ± SD. One-way ANOVA and Bonferroni test.

Asterisks indicate values significantly different from control (^∗∗∗^p < 0.001 and ^**^p < 0.01) and n.s. indicates not significantly different from the control. n is denoted below the bars.

**
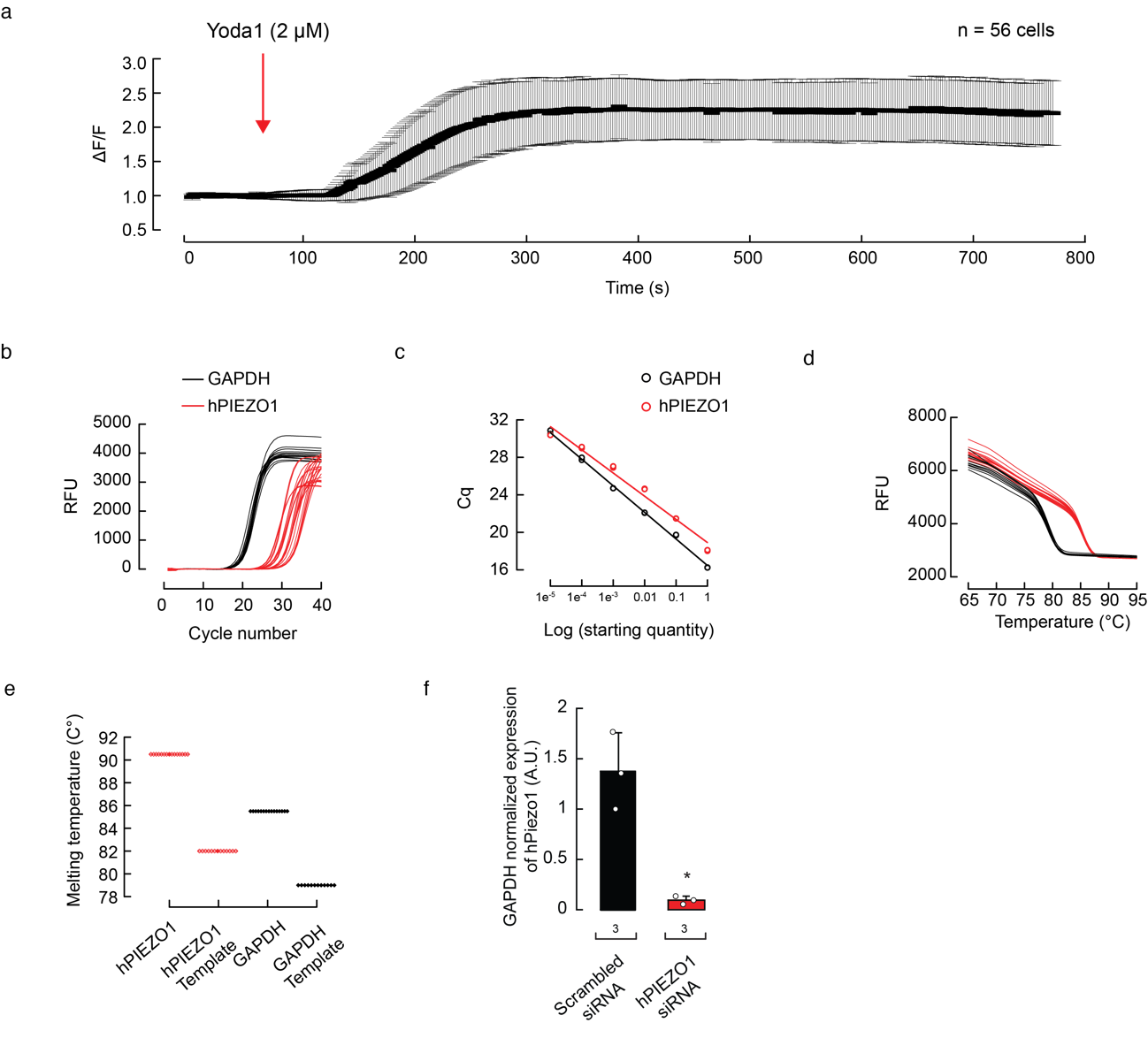
**

**Supplementary figure 5. Related to figure 4.**

**a** Mean intensity values (ΔF/F) of HMVEC perfused with control solution and Yoda1. Squares are mean ± SD. n is denoted on top of the graph.

**b** Relative fluorescence units (RFU) for HMVEC samples at every cycle of one RT-qPCR. Each trace represents data from a single fluorophore in one well.

**c** Standard curves showing the quantification as a function of the log of the starting quantity (R^2^ for housekeeping gene GAPDH = 0.998 and for hPIEZO1 = 0.976).

**d** Melting curves showing RFU as a function of temperature for each well.

**e** Box plot depicting the melt peak values for each experimental sample.

**f** Relative normalized expression of hPIEZO1 (ΔΔCq) for HMVEC transfected with scrambled siRNA and hPIEZO1 siRNA, respectively. Bars are mean ± SD. n is denoted inside bars. Unpaired t-test with Welch correction.

Asterisks indicate values significantly different from control (^∗^p < 0.05).

**
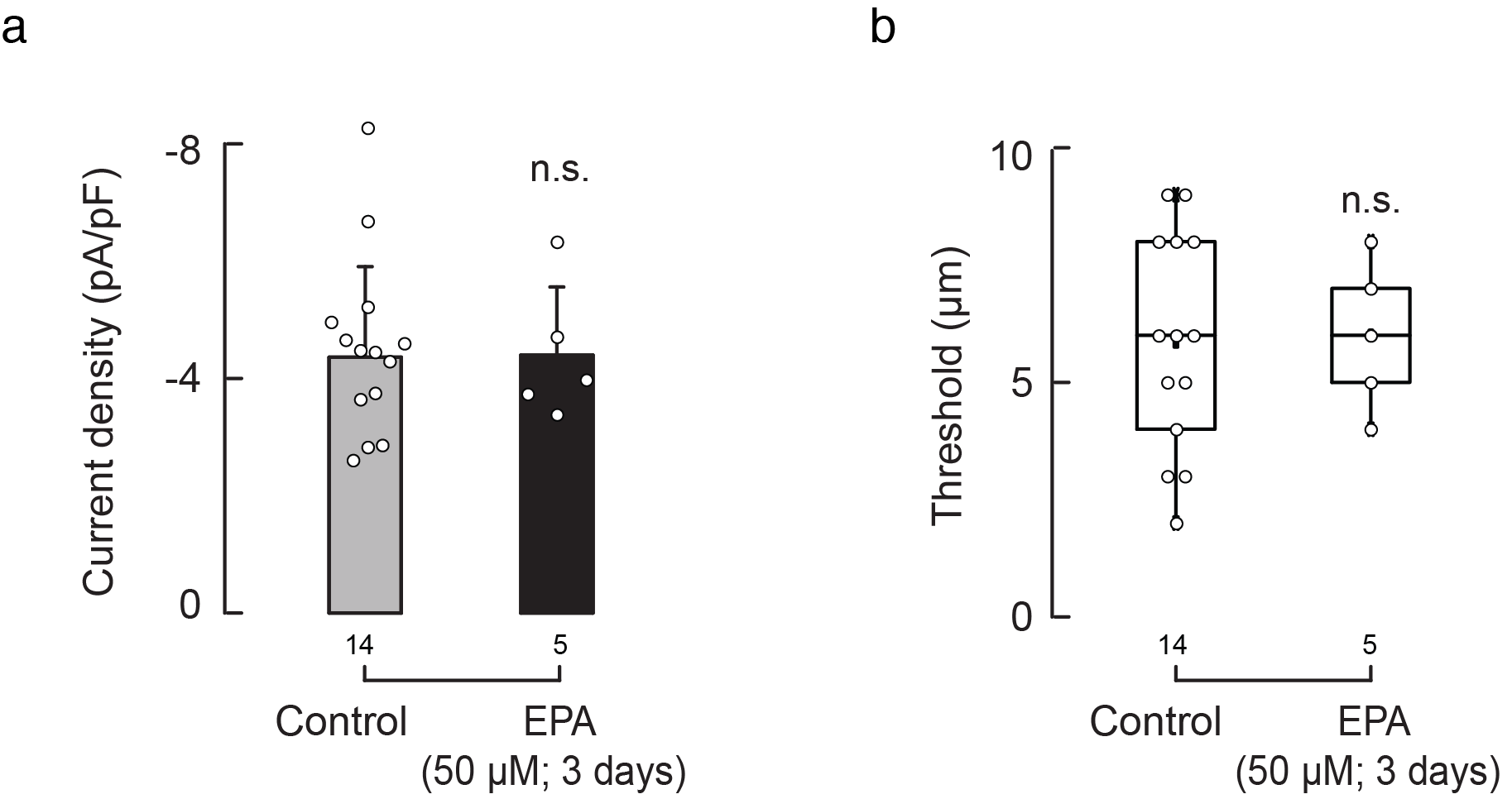
**

**Supplementary figure 6. Related to figure 5.**

**a** Piezo1 current densities elicited by maximum displacement of control and EPA (50 µM each day for three days)-treated HMVEC. Bars are mean ± SD. Unpaired t-test.

**b** Boxplots show the mean, median, and the 75^th^ to 25^th^ percentiles of the displacement threshold required to elicit Piezo1 currents of control and EPA (50 µM each day for three days)-treated HMVEC. Unpaired t-test.

n.s. indicates not significantly different from the control. n is denoted above the *x*-axes.

**
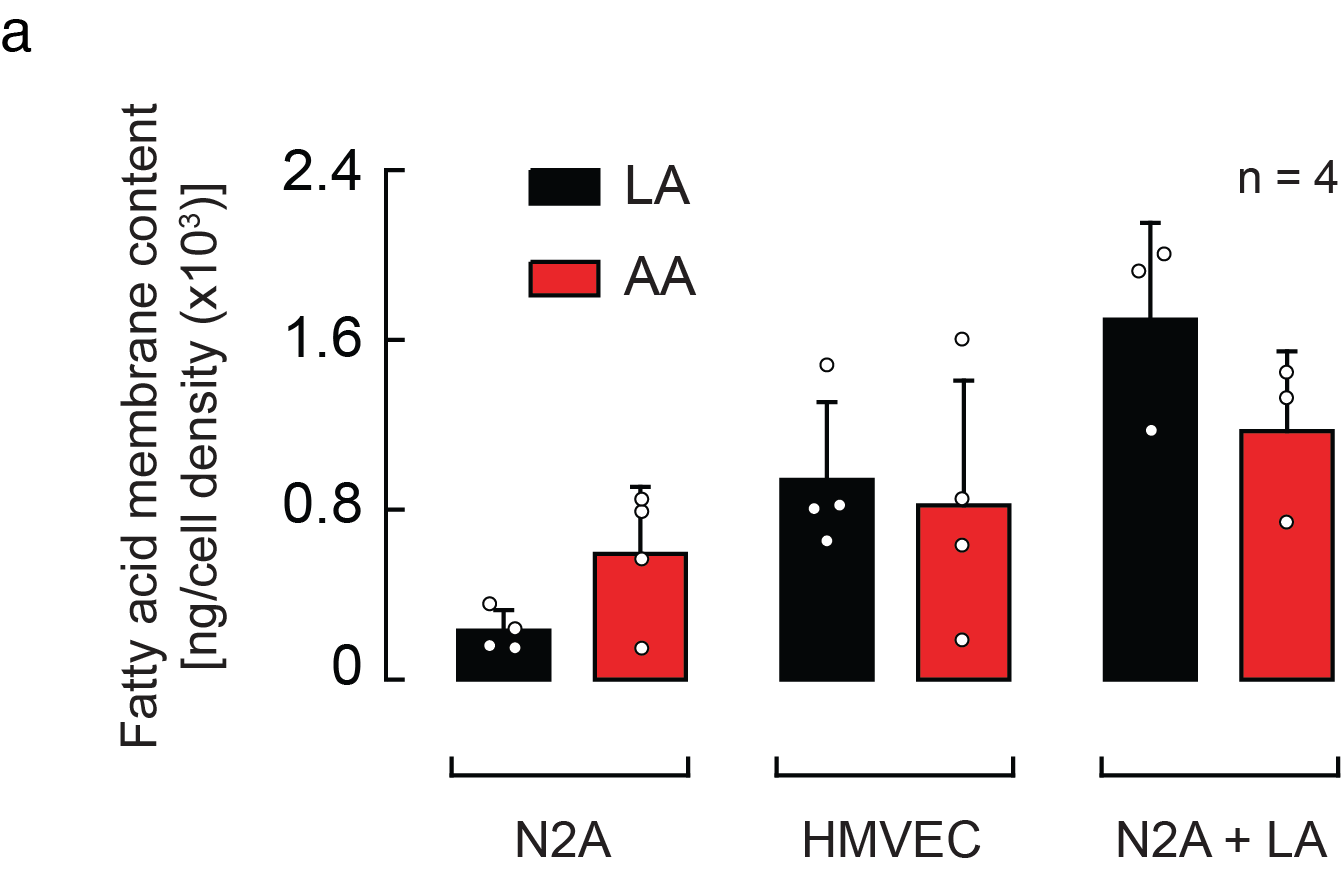
**

**Supplementary figure 7. Related to figure 6.**

**a** Linoleic acid (LA) and AA membrane content in N2A cells, HMVEC, and LA (100 μM)-treated N2A cells, as determined by LC-MS. n is denoted above the graph.


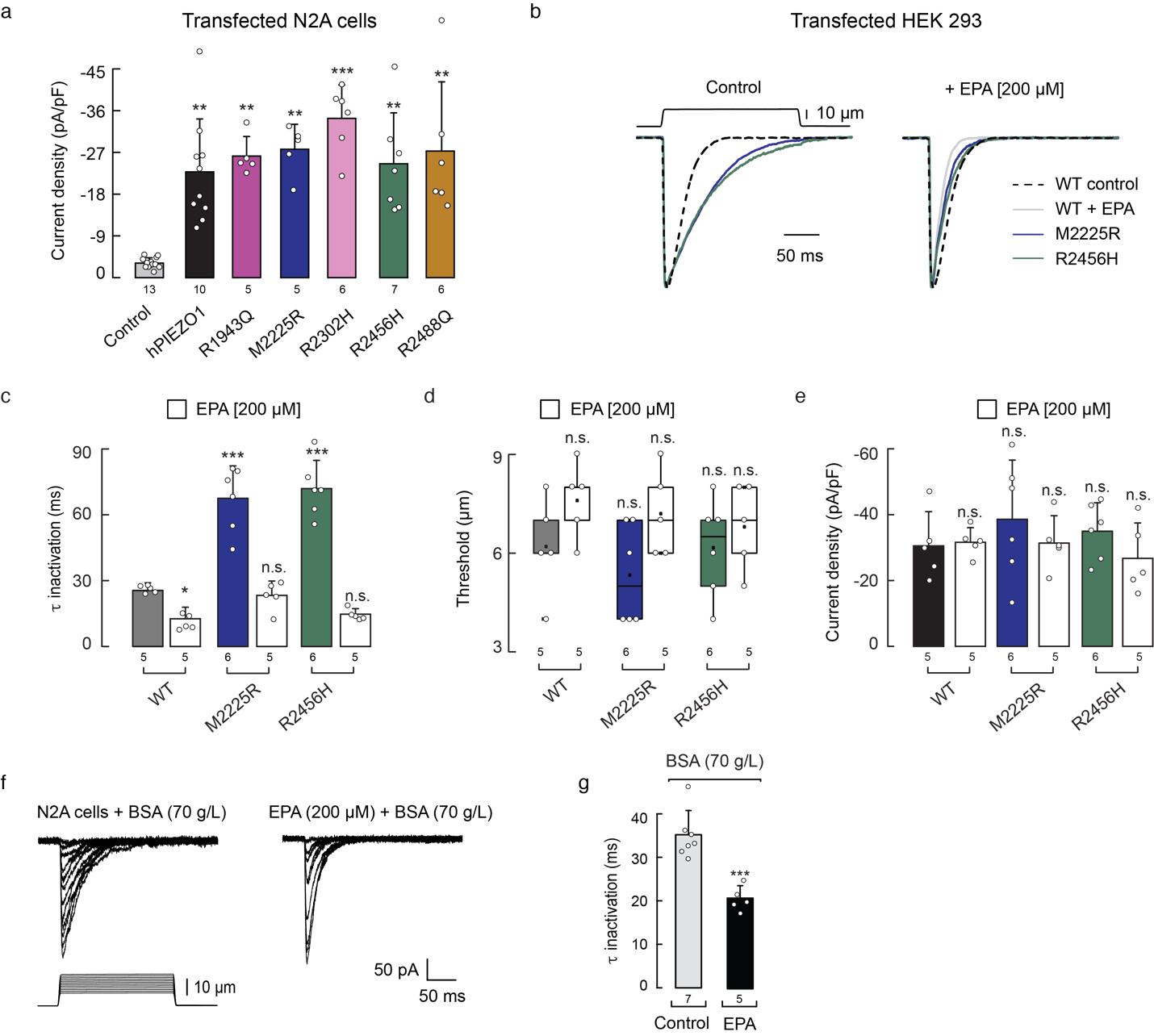


**Supplementary figure 8. Related to figure 7 and 8.**

**a** Current densities elicited by maximum displacement of wild type (WT) and xerocytosis mutationa R1943Q, M2225R, R2302H, R2456H, and R2488Q transfected N2A cells. Bars are mean ± SD. Kruskal-Wallis and Dunn’s multiple comparisons test.

**b** Representative normalized macroscopic currents (at -60 mV) evoked by maximum displacement of HEK-293 cells transfected with human Piezo1 wild type (WT) and xerocytosis mutations M2225R and R2456H with and without EPA (200 µM) supplementation.

**c** Piezo1 time constants of inactivation elicited by maximum displacement of WT and xerocytosis mutations M2225R and R2456H (transfected in HEK-293 cells) with and without EPA (200 µM) supplementation. Bars are mean ± SD. One-way ANOVA with Bonferroni test.

**d** Boxplots show the mean, median, and the 75^th^ to 25^th^ percentiles of the displacement threshold required to elicit Piezo1 currents of WT and xerocytosis mutations M2225R and R2456H (transfected in HEK-293 cells) with and without EPA (200 µM) supplementation. One-way ANOVA with Bonferroni test.

**e** Current densities elicited by maximum displacement of WT and xerocytosis mutations M2225R and R2456H (transfected in HEK-293 cells) with and without EPA (200 µM) supplementation. One-way ANOVA with Bonferroni test.

**f** Representative whole-cell patch-clamp recordings (at -60 mV) of N2A cells treated with BSA (70 g/L) with and without EPA (200 µM) supplementation elicited by mechanical stimulation.

**g** Piezo1 time constants of inactivation elicited by maximum displacement of N2A cells treated with BSA (70 g/L) with and without EPA (200 µM) supplementation. Bars are mean ± SD. Unpaired t-test.

Asterisks indicate values significantly different from control (^∗∗∗^p < 0.001, ^∗∗^p < 0.01, and ^∗^p < 0.05). n is denoted above the *x-*axes.
