# Supplementary material for: Dietary fatty acids fine-tune Piezo1 mechanical response
Source: Nat Commun. 2019 Mar 13;10:1200. doi: 10.1038/s41467-019-09055-7 (PMC6416271; doi:10.1038/s41467-019-09055-7)
Supplement: Supplementary file 3 — Reporting Summary [file 41467_2019_9055_MOESM3_ESM.pdf]

nature research

Corresponding author(s): NCOMMS-18-30385

Reporting Summary

Nature Research wishes to improve the reproducibility of the work that we publish. This form provides structure for consistency and transparency in reporting. For further information on Nature Research policies, see Authors & Referees and the Editorial Policy Checklist.

Statistical parameters

When statistical analyses are reported, confirm that the following items are present in the relevant location (e.g. figure legend, table legend, main text, or Methods section).

n/a	Confirmed	
☐	☒	The exact sample size (*n*) for each experimental group/condition, given as a discrete number and unit of measurement
☐	☒	An indication of whether measurements were taken from distinct samples or whether the same sample was measured repeatedly
☐	☒	The statistical test(s) used AND whether they are one- or two-sided<br>*Only common tests should be described solely by name; describe more complex techniques in the Methods section.*
☐	☒	A description of all covariates tested
☐	☒	A description of any assumptions or corrections, such as tests of normality and adjustment for multiple comparisons
☐	☒	A full description of the statistics including central tendency (e.g. means) or other basic estimates (e.g. regression coefficient) AND variation (e.g. standard deviation) or associated estimates of uncertainty (e.g. confidence intervals)
☒	☐	For null hypothesis testing, the test statistic (e.g. *F*, *t*, *r*) with confidence intervals, effect sizes, degrees of freedom and *P* value noted<br>*Give P values as exact values whenever suitable.*
☒	☐	For Bayesian analysis, information on the choice of priors and Markov chain Monte Carlo settings
☒	☐	For hierarchical and complex designs, identification of the appropriate level for tests and full reporting of outcomes
☒	☐	Estimates of effect sizes (e.g. Cohen's *d*, Pearson's *r*), indicating how they were calculated
☐	☒	Clearly defined error bars<br>*State explicitly what error bars represent (e.g. SD, SE, CI)*

Our web collection on statistics for biologists may be useful.

Software and code

Policy information about availability of computer code

Data collection	For electrophysiology: Clampex v10.4.2.0 (Molecular Devices, LLC); for atomic force microscopy (AFM): NanoScope v9.30 (Bruker Corporation), for Ca2+ imaging: CellSens Dimension v1.18 (OLYMPUS Corp.), for qRT-PCR: Bio-Rad CFX Maestro 1.0 v4.0.2325.0418 (Bio-Rad Laboratories), and for liquid chromatography mass spectrometry (LC-MS) we used the services of the NIH-funded facility at Wayne State University.
Data analysis	For electrophysiology: Clampfit v10.4.2.0 (Molecular Devices, LLC);FM: NanoScope v9.30 (Bruker Corporation), for Ca2+ imaging: CellSens Dimension v1.18 (OLYMPUS Corp.); for qRT-PCR:Bio-Rad CFX Maestro 1.0 v4.0.2325.0418 (Bio-Rad Laboratories)

For manuscripts utilizing custom algorithms or software that are central to the research but not yet described in published literature, software must be made available to editors/reviewers upon request. We strongly encourage code deposition in a community repository (e.g. GitHub). See the Nature Research guidelines for submitting code & software for further information.

Data

Policy information about availability of data

All manuscripts must include a data availability statement. This statement should provide the following information, where applicable:

- Accession codes, unique identifiers, or web links for publicly available datasets
- A list of figures that have associated raw data
- A description of any restrictions on data availability

The datasets generated during and/or analyzed during the current study are available from the corresponding author on reasonable request. Source data will be available upon publication: 10.6084/m9.figshare.7710140

Field-specific reporting

Please select the best fit for your research. If you are not sure, read the appropriate sections before making your selection.

☒ Life sciences ☐ Behavioural & social sciences ☐ Ecological, evolutionary & environmental sciences

For a reference copy of the document with all sections, see nature.com/authors/policies/ReportingSummary-flat.pdf

Life sciences study design

All studies must disclose on these points even when the disclosure is negative.

Sample size	No sample-size calculation was performed. For electrophysiology: we obtained data for more than 4 samples per condition (at least three different days). For AFM: we obtained more than 20 measurements, from at least two different tissue-culture plates. For Ca2+ imaging: we obtained data from 5 cover-slips, on three different days. For differential scanning calorimetry (DSC): we obtained data from 2 independent liposome samples, on two different days – the difference between measurements is less than 0.01. For LC-MS: we obtained fatty acid profiles from an ensemble of 2 million cells per condition.
Data exclusions	For electrophysiological experiments we excluded from the analyses (exclusion criteria were pre-established): (1) cells with leak currents bigger than 200 pA, (2) cells that detached from the substrate during mechanical stimulation and (3) cells which giga-seals did not withstand at least 6 indentation steps of the protocol. For Ca2+ imaging experiments, cells with a large baseline fluorescence signal (> 20 arbitrary units) were excluded from the analysis. For AFM, we excluded data points larger than 200 pN. For DSC, LC-MS, and RT-qPCR experiments we did not exclude data from the analyses.
Replication	All attempts at replication were successful. Electrophysiology, Ca2+ imaging, and RT-qPCR experiments were performed at least 3 times in different days with different preparations. DSC experiments were performed twice with independent preparations. Mass spec experiments were performed four times for N2A cells and HMVEC to determine fatty acids distribution, and three times for N2A cells supplemented with linoleic acid; cells suplemented with different fatty acids were measured once along with their control.
Randomization	Daily measurements included the control and treated samples. For electrophysiological experiments transfected cells had a fluorescent transfection marker.
Blinding	The investigators were not blind during data acquisition and analysis.

Reporting for specific materials, systems and methods

Materials & experimental systems

n/a	Involved in the study
☒	☐ Unique biological materials
☒	☐ Antibodies
☐	☒ Eukaryotic cell lines
☒	☐ Palaeontology
☒	☐ Animals and other organisms
☒	☐ Human research participants

Methods

n/a	Involved in the study
☒	☐ ChIP-seq
☒	☐ Flow cytometry
☒	☐ MRI-based neuroimaging

Eukaryotic cell lines

Policy information about cell lines

Cell line source(s)	Neuro-2a (N2A, ATCC CCL-131) and human embryonic kidney (HEK-293ATCC CRL-1573) cells from ATCC and Primary human microvascular endothelial cells (HMVEC, CSC 2M1) from Cell Systems
Authentication	None of the cell lines used were authenticated in the lab. However, N2A and HEK-293 cells were directly purchased from ATCC. HMVEC, from Cell Systems are authenticated by short tandem repeat (STR) profiling by ATCC.
Mycoplasma contamination	All cell lines tested negative for mycoplasma contamination.
Commonly misidentified lines (See ICLAC register)	*Name any commonly misidentified cell lines used in the study and provide a rationale for their use.*
